# Supplementary material for: When should lockdown be implemented? Devising cost-effective strategies for managing epidemics amid vaccine uncertainty
Source: PLoS Comput Biol. 2024 Jul 18;20(7):e1012010. doi: 10.1371/journal.pcbi.1012010 (PMC11288439; doi:10.1371/journal.pcbi.1012010)
Supplement: S1 Text — Contains further information regarding the epidemiological model structure and parameters. Contains additional sensitivity analysis figures. (PDF) [file pcbi.1012010.s001.pdf]

Model Description

Here we present a detailed description of the model used to simulate disease dynamics as briefly outlined in the main text. The population size is $N = 1$ million, disaggregated by three demographic cohorts $G = \{0-19,\ 20-64,\ 65+\}$ of sizes $N_{0-19} = 233,000$, $N_{20-64} = 580,000$, $N_{65+} = 187,000$ to resemble a subset of the United Kingdom demography structure [1].

Individuals who are susceptible (S_a) can acquire infection from infectious individuals and become exposed ($E_{a,m}$) before progressing to the infectious compartments upon the completion of the latent period. The model distinguishes between four infectious compartments per age cohort. Infectious individuals can have either symptomatic infection with age-dependent probability d_a or asymptomatic ($I^A_{a,m}$) infection without developing symptoms with probability $1 - d_a$. Symptomatic infection is further subdivided into compartments for individuals who are symptomatic without requiring hospital treatment ($I^S_{a,m}$) with age-dependent probability $d_a(1 - h_a)$, symptomatic who will inevitably require hospital treatment ($I^{PH}_{a,m}$) with probability $d_a h_a$ or individuals in hospital with severe disease (I^H_a), who have progressed from the "pre-hospitalisation" compartment ($I^{PH}_{a,m}$) at rate γ. All infectious classes contribute towards pathogen transmission, with reduced levels of infectiousness τ assumed for asymptomatic individuals in comparison to symptomatic individuals. Additionally, it is assumed that individuals in the pre-hospital compartment carry the same contact rate as individuals with general symptomatic infection, but hospitalised individuals are less infectious by a factor ρ due to ward isolation. Individuals in hospital (who avoid disease-induced mortality with age-dependent probability $1 - \mu_a$) recover at a reduced rate $\delta < \gamma$. The final consideration to the model structure is to incorporate waning immunity and the possibility of reinfection. Hence, a recovered class $R_{a,m}$ is modelled explicitly where individuals progress to upon the completion of the infectious stages. Recovered individuals remain in the class until immunity wanes at rates ω and they return to susceptibility, this rate is chosen to be sufficiently small as to not overestimate the number of reinfections in the early stages of the outbreak. It should be noted that once individuals return to the susceptible compartment, the probability of severe disease is independent of previous infection status.

We apply the method of stages to ensure that the latent, infectious and recovered periods are gamma distributed as opposed to exponentially distributed in the equivalent stochastic framework. To enforce a latent period with a total length ϵ^{-1} which is approximately Erlang distributed, there are $M = 3$ exposed compartments ($E_{a,1}$, $E_{a,2}$, $E_{a,3}$) for individuals who have been infected but are not yet infectious. Similarly, there are M compartments each for asymptomatic infection, symptomatic infection and recovery ($I^A_{a,m}$, $I^S_{a,m}$, $I^{PH}_{a,m}$, $R_{a,m}$ with $m \in \{1, 2, 3\}$) such that the rates for leaving these classes γ^{-1} and ω^{-1} are Erlang distributed with shape $M = 3$ in the equivalent stochastic framework. The exception is the hospitalisation stage which is constrained to a single compartment I^H_a. The length of the hospitalisation period δ^{-1} would be exponentially distributed if modelled explicitly. The length of stay in hospital follows a distribution which is approximately

exponential and the choice of δ parameter was decided by fitting an exponential curve to this distribution. Due to the short simulation timescales of this study (on the order of 2-3 years) and the broad size of the age bins, the model does not account for natural births, deaths and ageing between demographic cohorts. The model dynamics are governed by a set of coupled ordinary differential equations (ODEs) for each demographic cohort $a \in G$, for $M = 3$ and $m \in \{2, 3\}$:

$$\begin{aligned}
\frac{dS_a}{dt} &= -\frac{S_a \lambda_a}{N_a} + M\omega R_{a,M}, \\
\frac{dE_{a,1}}{dt} &= \frac{S_a \lambda_a}{N_a} - M\epsilon E_{a,1}, \\
\frac{dE_{a,m}}{dt} &= M\epsilon (E_{a,m-1} - E_{a,m}), \\
\frac{dI^A_{a,1}}{dt} &= M\epsilon (1 - d_a) E_{a,M} - M\gamma I^A_{a,1}, \\
\frac{dI^A_{a,m}}{dt} &= M\gamma (I^A_{a,m-1} - I^A_{a,m}), \\
\frac{dI^S_{a,1}}{dt} &= M\epsilon d_a (1 - h_a) E_{a,M} - M\gamma I^S_{a,1}, \\
\frac{dI^S_{a,m}}{dt} &= M\gamma (I^S_{a,m-1} - I^S_{a,m}), \\
\frac{dI^{PH}_{a,1}}{dt} &= M\epsilon d_a h_a E_{a,M} - M\gamma I^{PH}_{a,1}, \\
\frac{dI^{PH}_{a,m}}{dt} &= M\gamma (I^{PH}_{a,m-1} - I^{PH}_{a,m}), \\
\frac{dI^H_a}{dt} &= M\gamma I^{PH}_{a,M} - \delta I^H_a, \\
\frac{dR_{a,1}}{dt} &= M\gamma (I^A_{a,M} + I^S_{a,M}) + (1 - \mu_a)\delta I^H_a \; - M\omega R_{a,1}, \\
\frac{dR_{a,m}}{dt} &= M\omega (R_{a,m-1} - R_{a,m}).
\end{aligned} \tag{1}$$

Transmission occurs in accordance to the age-specific force of infection,

$$\lambda_a = \sum_{b \in G} \beta_{ba} \Big(\sum_{m \in \{1,2,3\}} (\tau I^A_{b,m} + I^S_{b,m} + I^{PH}_{b,m}) + \rho I^H_b \Big). \tag{2}$$

The parameter β_{ba} captures transmission from individuals in cohort b to individuals in cohort a. Transmission is driven by an age-structured contact matrix π for the United Kingdom taken from Prem *et al.* [2]. Each entry π_{ij} is an aggregation of the number of contacts with age group j recorded by age group i.

$$\pi = \begin{pmatrix} 6.8045 & 4.0791 & 0.2121 \\ 1.9898 & 8.1466 & 0.55565 \\ 0.5221 & 3.4354 & 2.0034 \end{pmatrix}. \tag{3}$$

This matrix is scaled by a transmission factor $\Pi = \Pi(R_0)$ in order to obtain a transmission matrix β such that the basic reproduction number will be $R_0 = 3.0$ upon numerical evaluation using the Next Generation Matrix (NGM) approach [3]. An interpretation of the transmission factor is that $1/\Pi$ is the probability of a transmission event occurring given contact. The remaining model parameters are fixed and their description and values are provided in Table A.

$$\beta_{ij} = \frac{1}{\Pi}\pi_{ij}. \tag{4}$$

The implementation of a control intervention is modelled by adjusting all contact rates by the intensity of the control state $u(t)$ – equivalent to the relative reduction in transmission. This gives time-dependent contact rates,

$$\beta_{ij}(t) = (1 - u(t))\beta_{ij}. \tag{5}$$

The age-specific parameters d_a, h_a, μ_a in addition to the recovery rate from hospitalisation δ were originally modelled as distributions by Keeling *et al.* [4], which are publicly available. To apply the age-specific distributions to this study, they were aggregated from the original five-year age bands $G' = \{0{-}4, 5{-}9, \ldots, 95{-}100, 100{+}\}$ to match the three age cohorts G used in this study. By defining subsets of the relevant age bands $G'_{0-19} = \{0-4, \ldots, 15-19\}$, $G'_{20-64} = \{20-24, \ldots, 60-64\}$, $G'_{65+} = \{65-69, \ldots, 100+\}$, the probability of symptomatic infection d_a is computed as follows,

$$d_a = \sum_{g \in G'_a} N_{g,a} d'_g\,, \qquad a \in G, \tag{6}$$

where $N_{g,a}$ is the proportion of the population group a which reside in age group g (taken from [1]) and d'_g is the original distribution for age bands G' [4]. The same methodology is used to compute h_a, μ_a. The time spent in the hospitalised class (X, measured in days) is assumed to obey an exponential distribution with mean $1/\delta$. The rate for leaving hospital δ is inferred by fitting the cumulative distribution function $F_X(x) = 1 - e^{-\delta x}$ to the cumulative distribution time in hospital distribution from Keeling *et al.* [4] using nonlinear least squares.

Parameter	Description	Estimate	Units	Source
R_0	Basic reproduction number	3.0	N/A	Assumed
Π	Transmission scaling for β	17.9241	N/A	Computed
τ	Relative asymptomatic infectiousness	0.25	N/A	[4]
ρ	Relative infectiousness of hospitalised individuals	0.1	N/A	Assumed
ϵ	Latency progression rate	1/5.28	days^{-1}	[5]
d_{0-19}	Probability of developing symptoms (age 0-19)	0.0275	N/A	[4]
d_{20-64}	Probability of developing symptoms (age 20-64)	0.1350	N/A	[4]
d_{65+}	Probability of developing symptoms (age 65+)	0.5374	N/A	[4]
h_{0-19}	Probability of hospitalisation given symptoms (age 0-19)	0.1268	N/A	[4]
h_{20-64}	Probability of hospitalisation given symptoms (age 20-64)	0.1173	N/A	[4]
h_{65+}	Probability of hospitalisation given symptoms (age 65+)	0.2430	N/A	[4]
μ_{0-19}	Probability of death given hospitalisation (age 0-19)	0.0276	N/A	[4]
μ_{20-64}	Probability of death given hospitalisation (age 20-64)	0.0533	N/A	[4]
μ_{65+}	Probability of death given hospitalisation (age 65+)	0.1935	N/A	[4]
γ	Recovery rate from infection (outside of hospital)	1/5	days^{-1}	[6]
δ	Recovery rate from hospitalisation	1/8.78	days^{-1}	[4]
ω	Waning immunity rate	1/800	days^{-1}	Assumed

Table A: Disease parameters. Table of fixed parameters and their biological interpretation in the mathematical model (1). Sensitivity to assumed parameters R_0, ρ and ω are presented in Figures D, E, I.

Sensitivity analysis

Here we present a sensitivity analysis to model simulations for each strategy under varying model parameters. We vary the parameters γ, δ, τ, ρ, ω, R_0 in addition to a severity scaling $\hat{d}$ (such that all age-specific probabilities of developing symptoms become scaled $d_a \rightarrow \hat{d}d_a$). Note that upon adjusting each parameter (except when sensitivity to R_0 is explicitly examined), the transmission scaling parameter Π is adjusted to maintain a basic reproduction number of $R_0 = 3$. Figures A - G showcase varying simulation dynamics (grey) for each parameter in question, in comparison to the trajectory used in the main manuscript and parameter values in Table A displayed in red. In Figures H - J, the strategy costs are compared as each parameter is varied. Costs are divided between the two key contributions in the objective function: Burden (cumulative hospitalisations) and Stringency (cumulative intensity in interventions). Both costs are normalised against the worst outcome across strategies for the same parameter value, such that the worst strategy always has a normalised cost contribution of one. We find that varying biological parameters can lead to a different outbreak trajectory for the same strategy as defined by the switching thresholds. However,

the relative ranking of strategies in Burden and Stringency cost contributions is relatively consistent: under most choices of parameter values the Rapid control strategy yields the greatest cost in disease and the Suppression strategy yields the greatest cost in maintaining interventions.

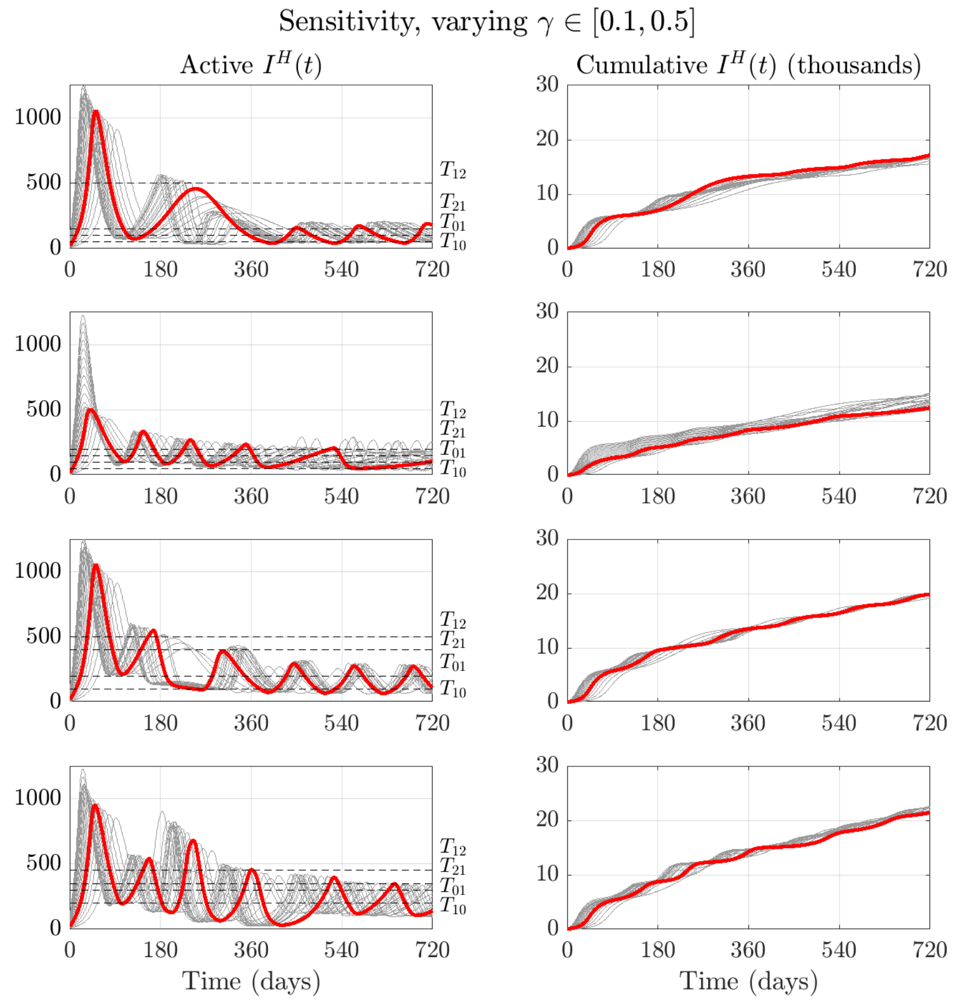

Figure A: Sensitivity to biological parameters (γ). Sensitivity in simulation dynamics for each control strategy (rows) in the scenario with no vaccination to varying select parameters $\gamma \in \{0.1, 0.12, \ldots, 0.5\}$ while keeping the remaining parameters fixed. Shown are the active hospitalised individuals across all age cohorts $I^H(t)$ for each strategy (left), and cumulative hospitalisations (right). Grey lines display the simulations under varying γ, with the red line representing the dynamics in the main text using parameter values from Table A. In the active hospitalisations subpanels, dashed lines are used to mark the values for switching thresholds T_{ij} used to move for control state i to control state j. The strategies appear in descending row order as follows: S1 (Cautious easing), S2 (Suppression), S3 (Slow control), S4 (Rapid control).

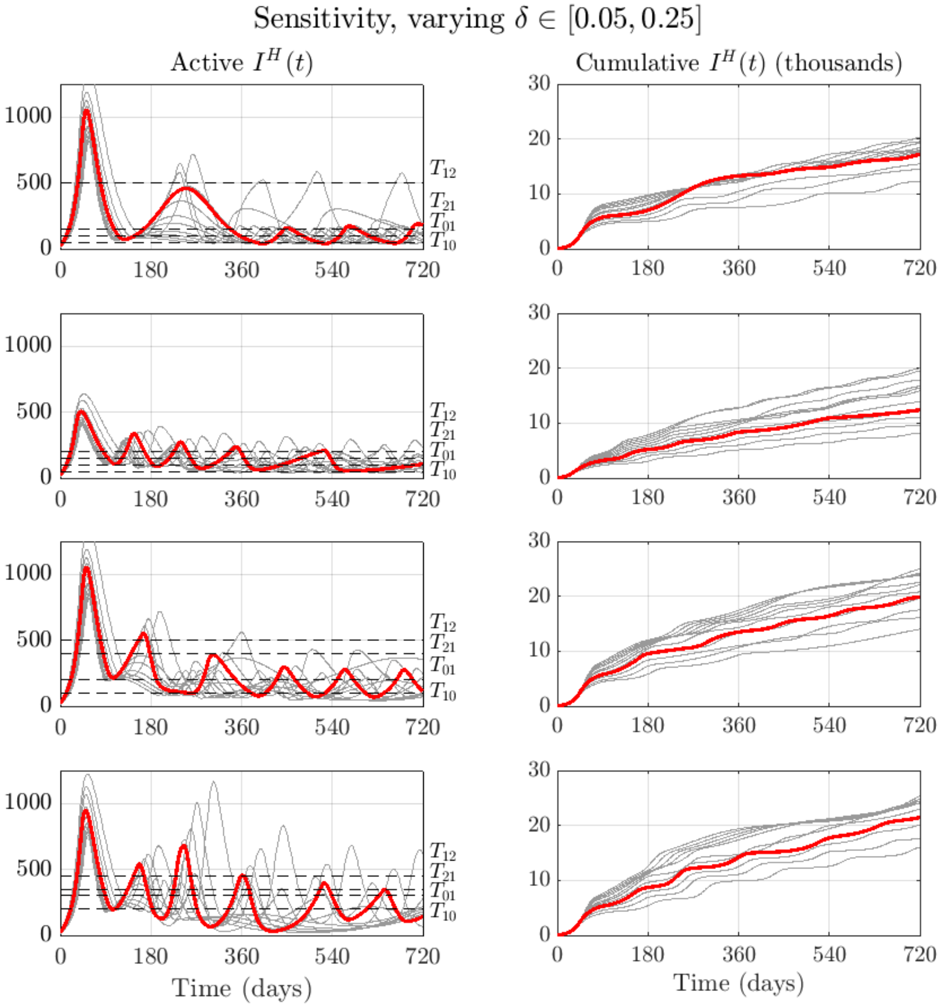

Figure B: Sensitivity to biological parameters (δ). Sensitivity in simulation dynamics for each control strategy (rows) in the scenario with no vaccination to varying select parameters $\delta \in \{0.05, 0.07, \ldots, 0.25\}$ while keeping the remaining parameters fixed. Shown are the active hospitalised individuals across all age cohorts $I^H(t)$ for each strategy (left), and cumulative hospitalisations (right). Grey lines display the simulations under varying δ, with the red line representing the dynamics in the main text using parameter values from Table A. In the active hospitalisations subpanels, dashed lines are used to mark the values for switching thresholds T_{ij} used to move for control state i to control state j. The strategies appear in descending row order as follows: S1 (Cautious easing), S2 (Suppression), S3 (Slow control), S4 (Rapid control).

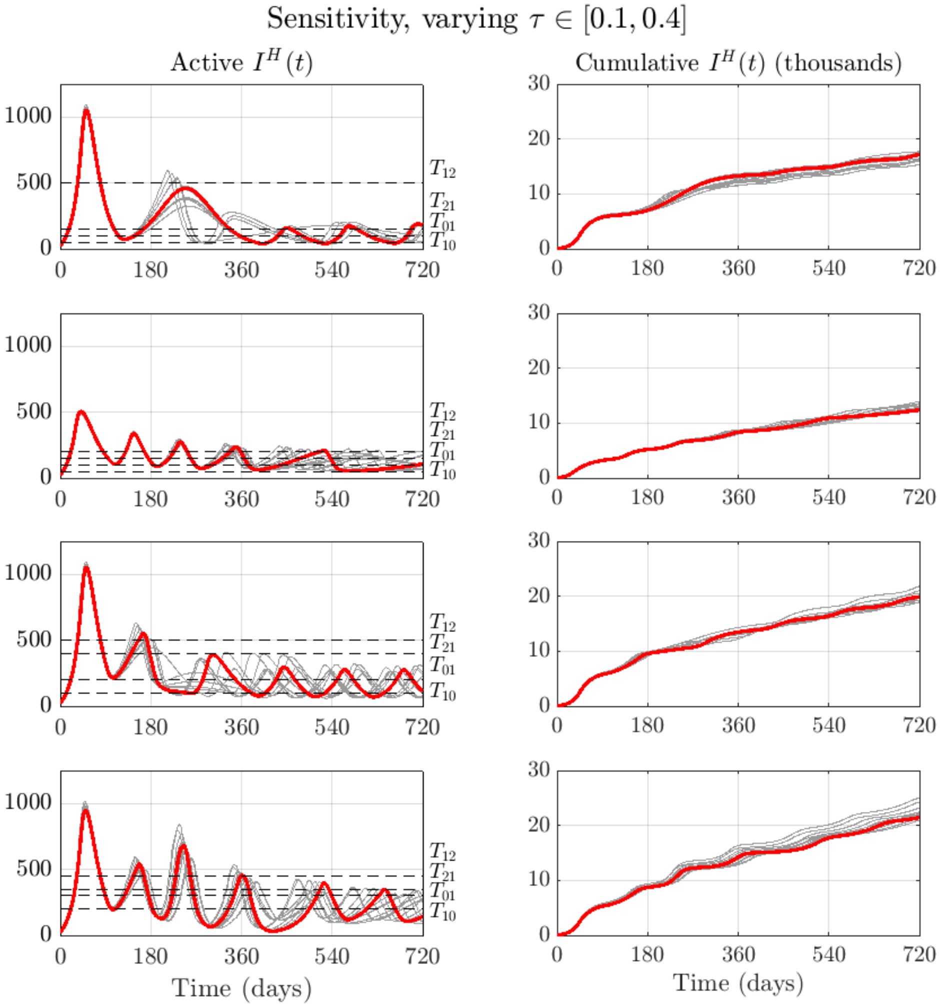

Figure C: Sensitivity to biological parameters (τ). Sensitivity in simulation dynamics for each control strategy (rows) in the scenario with no vaccination to varying select parameters $\tau \in \{0.1, 0.13, \ldots, 0.4\}$ while keeping the remaining parameters fixed. Shown are the active hospitalised individuals across all age cohorts $I^H(t)$ for each strategy (left), and cumulative hospitalisations (right). Grey lines display the simulations under varying τ, with the red line representing the dynamics in the main text using parameter values from Table A. In the active hospitalisations subpanels, dashed lines are used to mark the values for switching thresholds T_{ij} used to move for control state i to control state j. The strategies appear in descending row order as follows: S1 (Cautious easing), S2 (Suppression), S3 (Slow control), S4 (Rapid control).

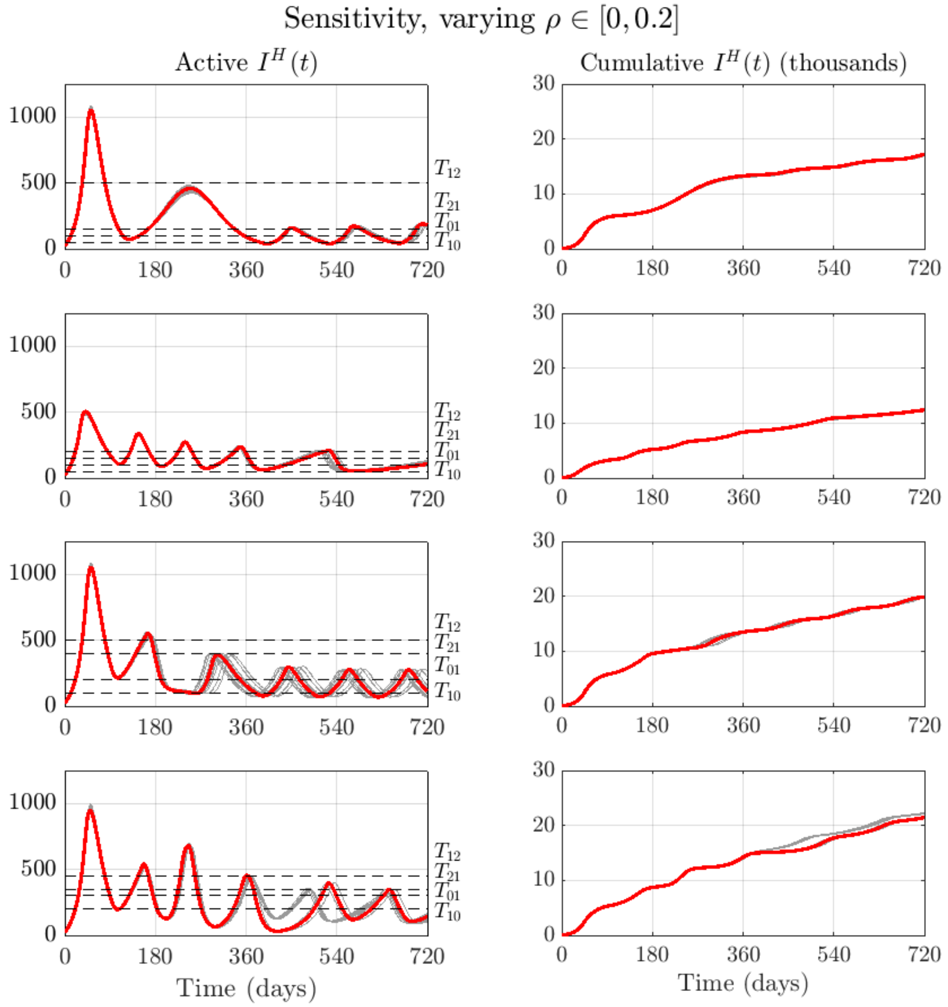

Figure D: Sensitivity to biological parameters (ρ). Sensitivity in simulation dynamics for each control strategy (rows) in the scenario with no vaccination to varying select parameters $\rho \in \{0, 0.02, \ldots, 0.2\}$ while keeping the remaining parameters fixed. Shown are the active hospitalised individuals across all age cohorts $I^H(t)$ for each strategy (left), and cumulative hospitalisations (right). Grey lines display the simulations under varying ρ, with the red line representing the dynamics in the main text using parameter values from Table A. In the active hospitalisations subpanels, dashed lines are used to mark the values for switching thresholds T_{ij} used to move for control state i to control state j. The strategies appear in descending row order as follows: S1 (Cautious easing), S2 (Suppression), S3 (Slow control), S4 (Rapid control).

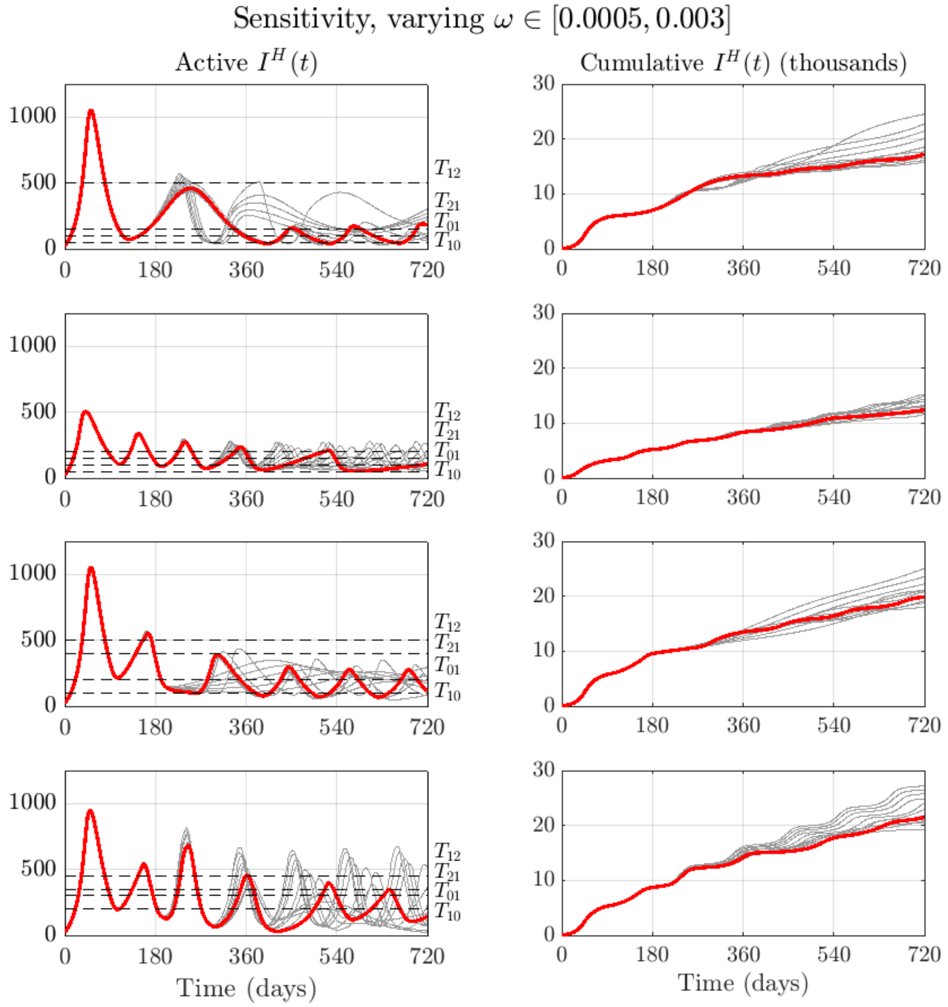

Figure E: Sensitivity to biological parameters (ω). Sensitivity in simulation dynamics for each control strategy (rows) in the scenario with no vaccination to varying select parameters $\omega \in \{0.0005, 0.00025, \ldots, 0.003\}$ while keeping the remaining parameters fixed. Shown are the active hospitalised individuals across all age cohorts $I^H(t)$ for each strategy (left), and cumulative hospitalisations (right). Grey lines display the simulations under varying ω, with the red line representing the dynamics in the main text using parameter values from Table A. In the active hospitalisations subpanels, dashed lines are used to mark the values for switching thresholds T_{ij} used to move for control state i to control state j. The strategies appear in descending row order as follows: S1 (Cautious easing), S2 (Suppression), S3 (Slow control), S4 (Rapid control).

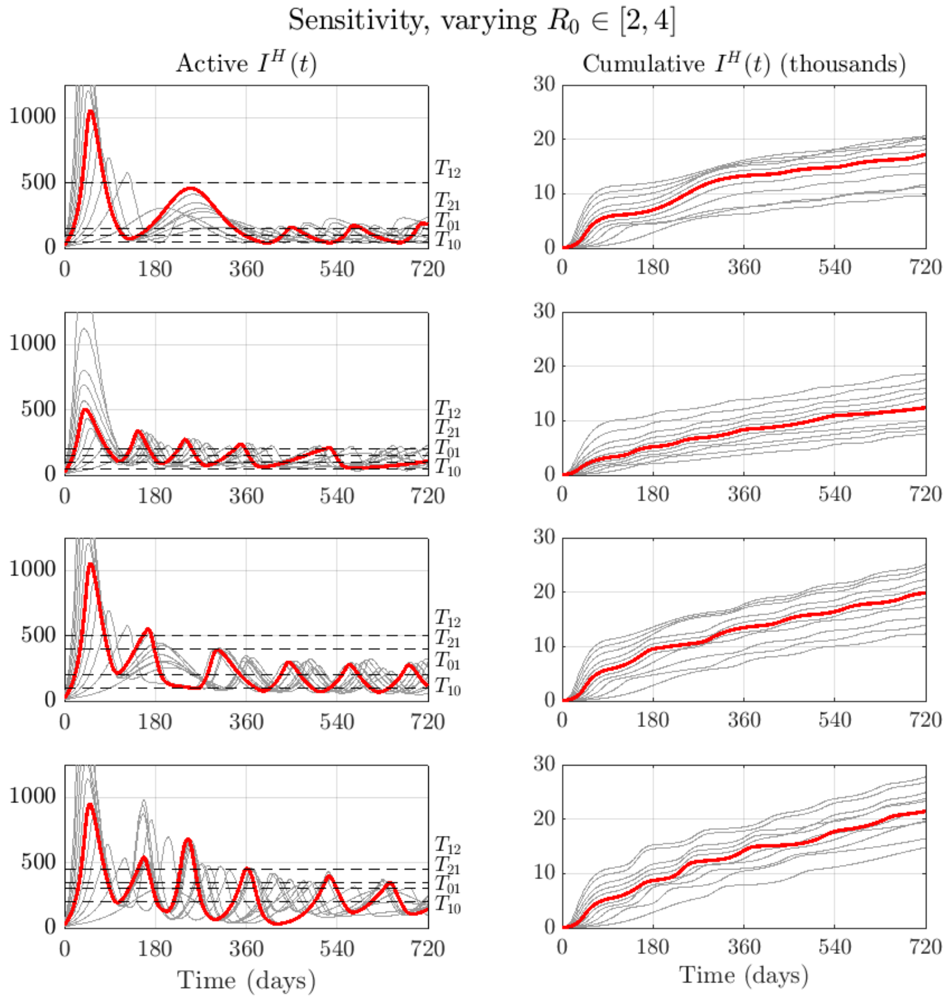

Figure F: Sensitivity to biological parameters (R_0). Sensitivity in simulation dynamics for each control strategy (rows) in the scenario with no vaccination to varying select parameters $R_0 \in \{2.0, 2.2, \ldots, 4.0\}$ while keeping the remaining parameters fixed. Shown are the active hospitalised individuals across all age cohorts $I^H(t)$ for each strategy (left), and cumulative hospitalisations (right). Grey lines display the simulations under varying R_0, with the red line representing the dynamics in the main text using parameter values from Table A. In the active hospitalisations subpanels, dashed lines are used to mark the values for switching thresholds T_{ij} used to move for control state i to control state j. The strategies appear in descending row order as follows: S1 (Cautious easing), S2 (Suppression), S3 (Slow control), S4 (Rapid control).

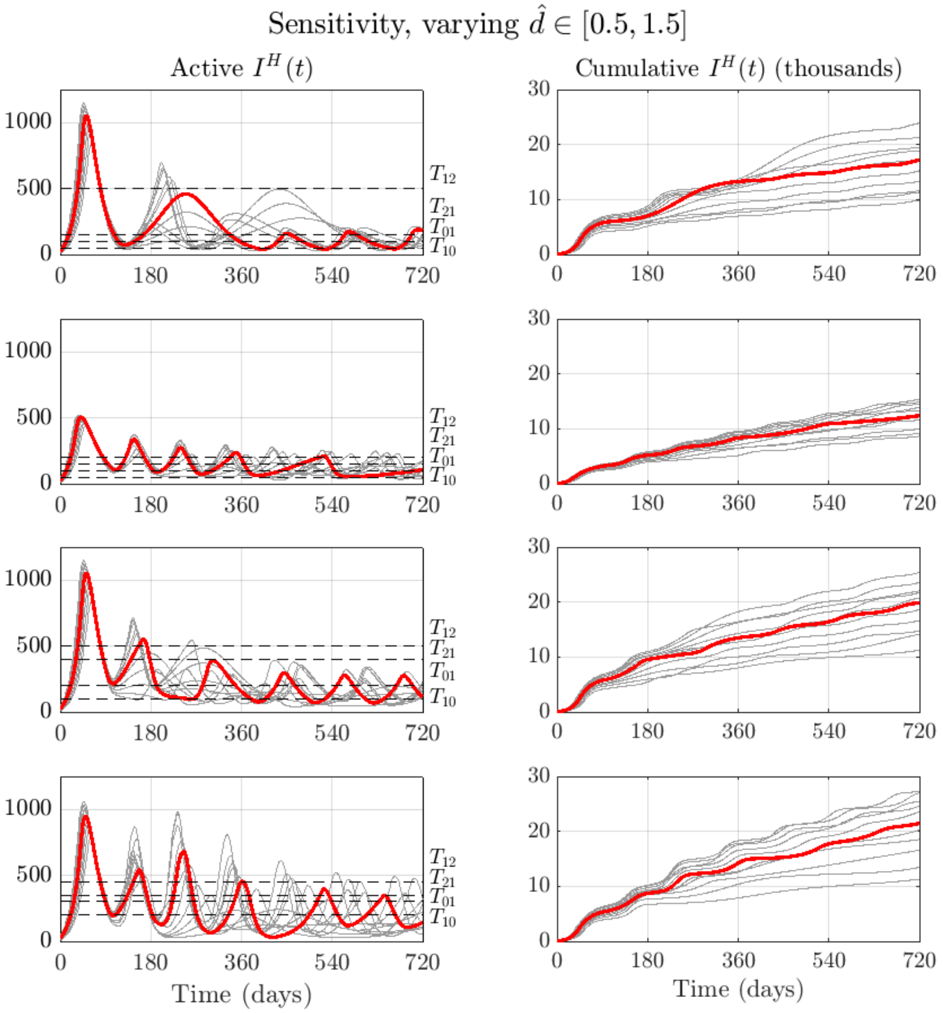

Figure G: Sensitivity to biological parameters ($\hat{d}$). Sensitivity in simulation dynamics for each control strategy (rows) in the scenario with no vaccination to varying select parameters $\hat{d} \in \{0.5, 0.6, \ldots, 1.5\}$ while keeping the remaining parameters fixed. This parameter $\hat{d}$ represents a severity scaling such that all age-specific probabilities of developing symptoms become scaled $d_a \to \hat{d}d_a$. Shown are the active hospitalised individuals across all age cohorts $I^H(t)$ for each strategy (left), and cumulative hospitalisations (right). Grey lines display the simulations under varying $\hat{d}$, with the red line representing the dynamics in the main text using parameter values from Table A. In the active hospitalisations subpanels, dashed lines are used to mark the values for switching thresholds T_{ij} used to move for control state i to control state j. The strategies appear in descending row order as follows: S1 (Cautious easing), S2 (Suppression), S3 (Slow control), S4 (Rapid control).

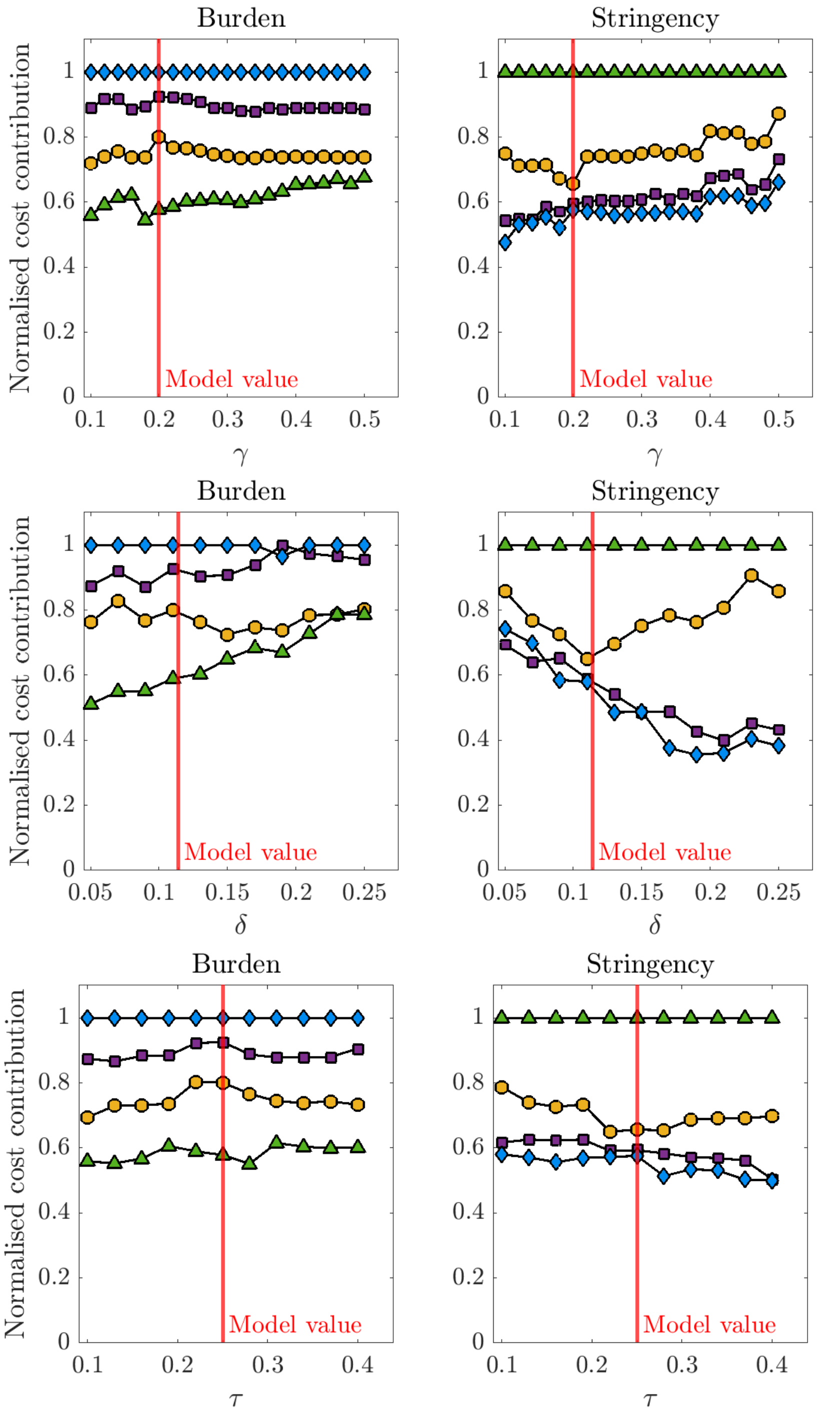

Figure H: Sensitivity to biological parameters (γ, δ, τ). Sensitivity in strategy cost contributions to varying select parameters γ, δ, τ (rows) while keeping the remaining parameters fixed. Columns display the two key contributions in the objective function: cumulative hospitalisations (left) and intensity of interventions (right), each normalised such that the worst strategy outcome has a normalised cost contribution of one for each choice of model parameter.

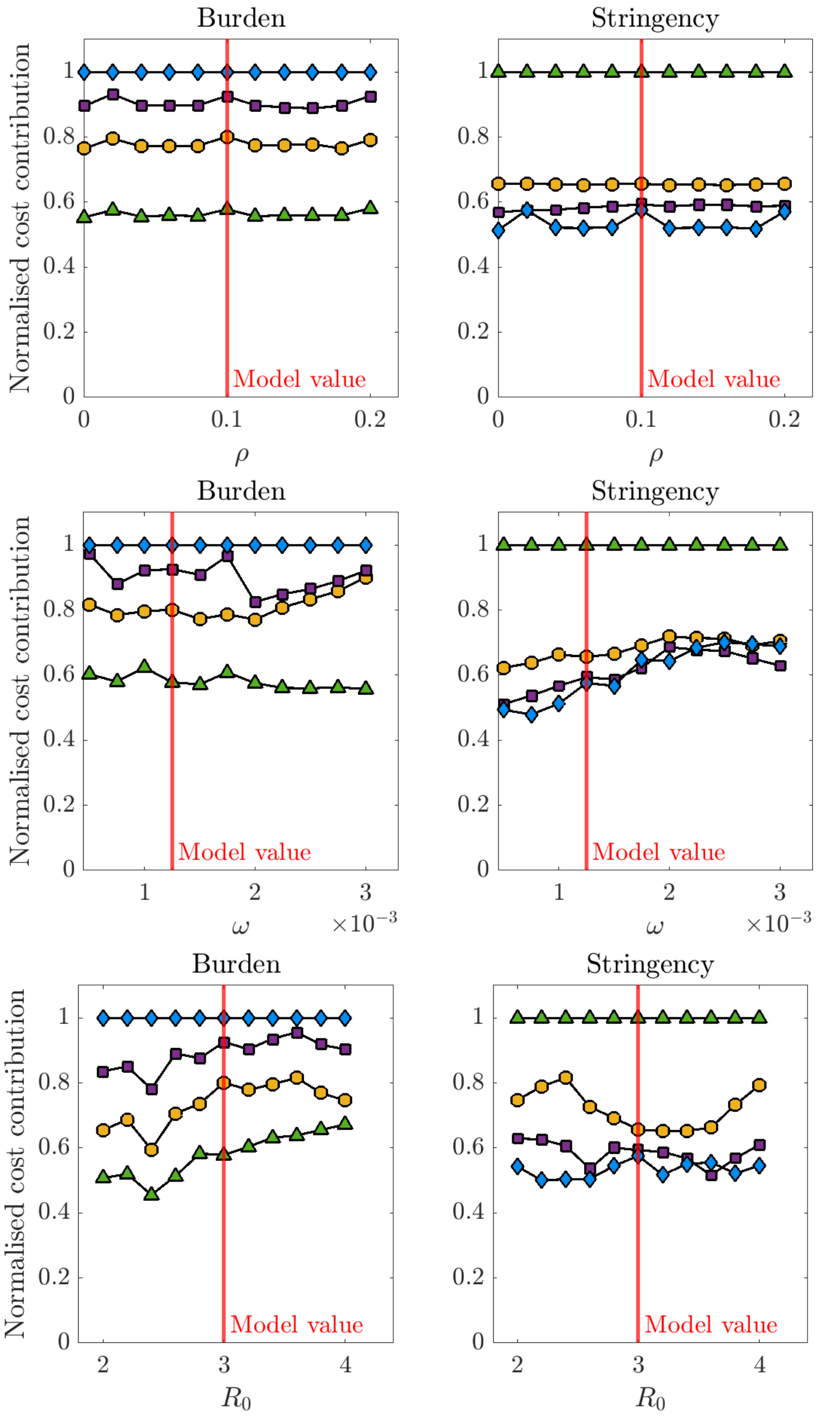

Figure I: Sensitivity to biological parameters (ρ, ω, R_0). Sensitivity in strategy cost contributions to varying select parameters ρ, ω, R_0 (rows) while keeping the remaining parameters fixed. Columns display the two key contributions in the objective function: cumulative hospitalisations (left) and intensity of interventions (right), each normalised such that the worst strategy outcome has a normalised cost contribution of one for each choice of model parameter.

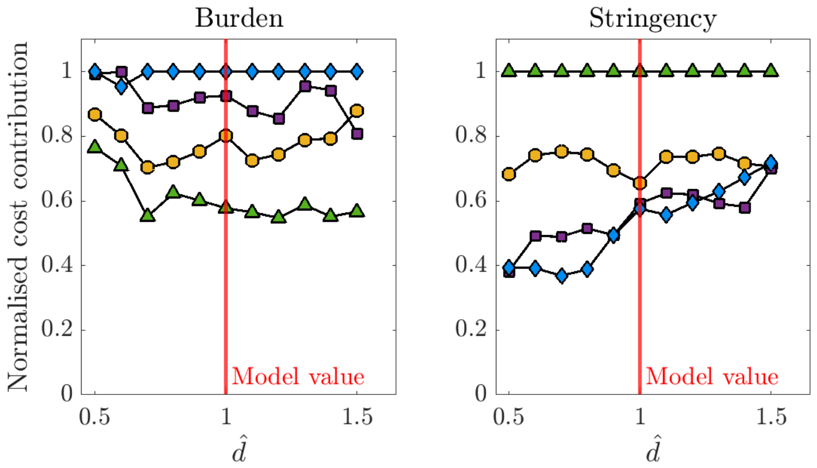

Figure J: Sensitivity to biological parameters ($\hat{d}$). Sensitivity in strategy cost contributions to varying select parameter $\hat{d}$ while keeping the remaining parameters fixed. This parameter $\hat{d}$ represents a severity scaling such that all age-specific probabilities of developing symptoms become scaled $d_a \to \hat{d}d_a$. Columns display the two key contributions in the objective function: cumulative hospitalisations (left) and intensity of interventions (right), each normalised such that the worst strategy outcome has a normalised cost contribution of one for each choice of model parameter.

References

1. Population Pyramid. United Kingdom 2020; 2023. Available from: `https://www.populationpyramid.net/united-kingdom/2020/`.

2. Prem K, Cook A, Jit M. Projecting social contact matrices in 152 countries using contact surveys and demographic data. PLoS Comput Bio. 2017;13(9):1–21. doi:10.1371/journal.pcbi.1005697.

3. Diekmann O, Heesterbeek J, Roberts M. The construction of next-generation matrices for compartmental epidemic models. J R Soc Interface. 2010;7(47):873–885. doi:10.1098/rsif.2009.0386.

4. Keeling M, Dyson L, Guyver-Fletcher G, Holmes A, Semple M, ISARIC4C Investigators, et al. Fitting to the UK COVID-19 outbreak, short-term forecasts and estimating the reproductive number. Stat Methods Med Res. 2022;31(9):1716–1737. doi:10.1177/09622802211070257.

5. Lauer S, Grantz K, Bi Q, Jones F, Zheng Q, Meredith H, et al. The Incubation Period of Coronavirus Disease 2019 (COVID-19) From Publicly Reported Confirmed Cases: Estimation and Application. Ann Intern Med. 2020;172(9):577–582. doi:10.7326/M20-0504.

6. Davies N, Kucharski A, Eggo R, Gimma A, Edmunds W. Effects of non-pharmaceutical interventions on COVID-19 cases, deaths, and demand for hospital services in the UK: a modelling study. Lancet Public Health. 2020;5(7):375–385. doi:10.1016/S2468-2667(20)30133-X.
