# Supplementary material for: When should lockdown be implemented? Devising cost-effective strategies for managing epidemics amid vaccine uncertainty
Source: PLoS Comput Biol. 2024 Jul 18;20(7):e1012010. doi: 10.1371/journal.pcbi.1012010 (PMC11288439; doi:10.1371/journal.pcbi.1012010)
Supplement: S2 Text — Clarifies the construction of vaccine marginal and joint probability distributions. (PDF) [file pcbi.1012010.s002.pdf]

Vaccination uncertainty

A total of four joint probability distributions were used to explore distributions in cost for each control strategy as the arrival date T and eventual coverage η of the vaccine varied. These were constructed by combining pairs of two marginal distributions each for T and η:

$$
\begin{aligned}
T &= 60 + 60Y, \\
1080 - T &= 60Y. \\
\eta &= 0.05Z, \\
1 - \eta &= 0.05Z,
\end{aligned}
\tag{1}
$$

where Y and Z are random variables defined over a sequence of integers $y = 0, 1, \ldots, 17$ and $z = 0, 1, \ldots, 20$ respectively. Their probability mass functions

$$
\begin{aligned}
\mathbb{P}[Y = y] &= e^{-k}\frac{k^y}{y!}/\Omega_Y, \\
\mathbb{P}[Z = z] &= e^{-l}\frac{l^z}{z!}/\Omega_Z,
\end{aligned}
\tag{2}
$$

are analogous to the Poisson distribution with rates $k = 6$ and $l = 5$, subject to normalising constants Ω_Y, Ω_Z to ensure that $\sum_y \mathbb{P}[Y = y] = 1 = \sum_z \mathbb{P}[Z = z]$. As showcased in S1 Fig, the Poisson distribution allows for sufficient variance across T, η outcomes for the marginal distributions while maintaining very low probabilities at the extremities $\mathbb{P}[T] = \{60, 1080\}$, $\mathbb{P}[\eta] = \{0, 1\}$ of the relevant sample space. The final joint probability distributions arising from these marginal distributions are displayed in S2 Fig.
