# Supplementary figures and images for: When should lockdown be implemented? Devising cost-effective strategies for managing epidemics amid vaccine uncertainty
Source: PLoS Comput Biol. 2024 Jul 18;20(7):e1012010. doi: 10.1371/journal.pcbi.1012010 (PMC11288439; doi:10.1371/journal.pcbi.1012010)

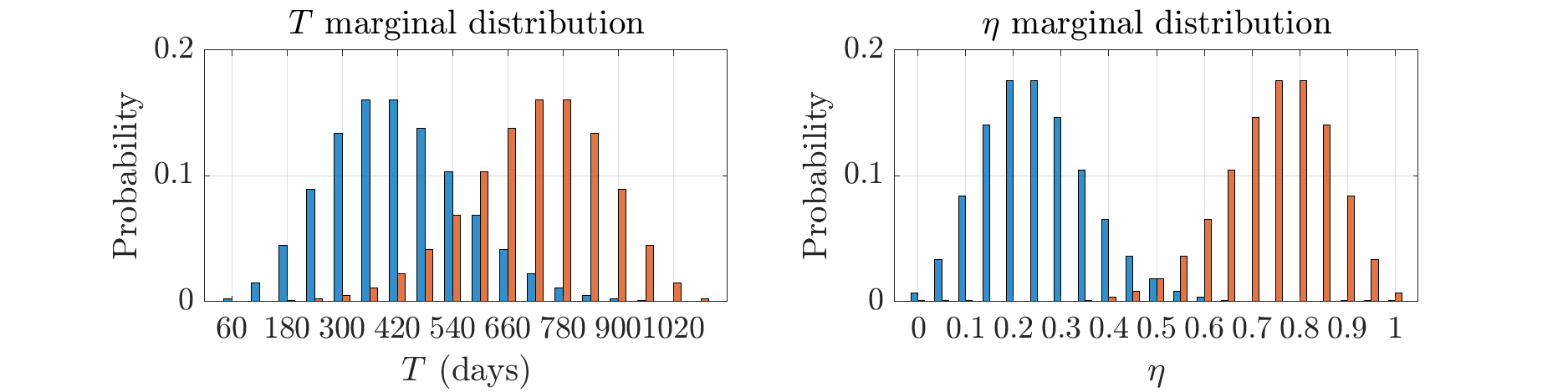

Supplement: S1 Fig — Marginal probability distributions for the vaccine deployment date T (left) and eventual coverage η (right). The distributions are generated using the random variables Y, Z who arise from discrete normal distributions with respective parameterisation (μT,σT2) and (μη,ση2). Different expectations for T, η are driven by varying μT, μη and the underlying variance is fixed, σT2=ση2=2.4. (TIF) [file pcbi.1012010.s003.tif]

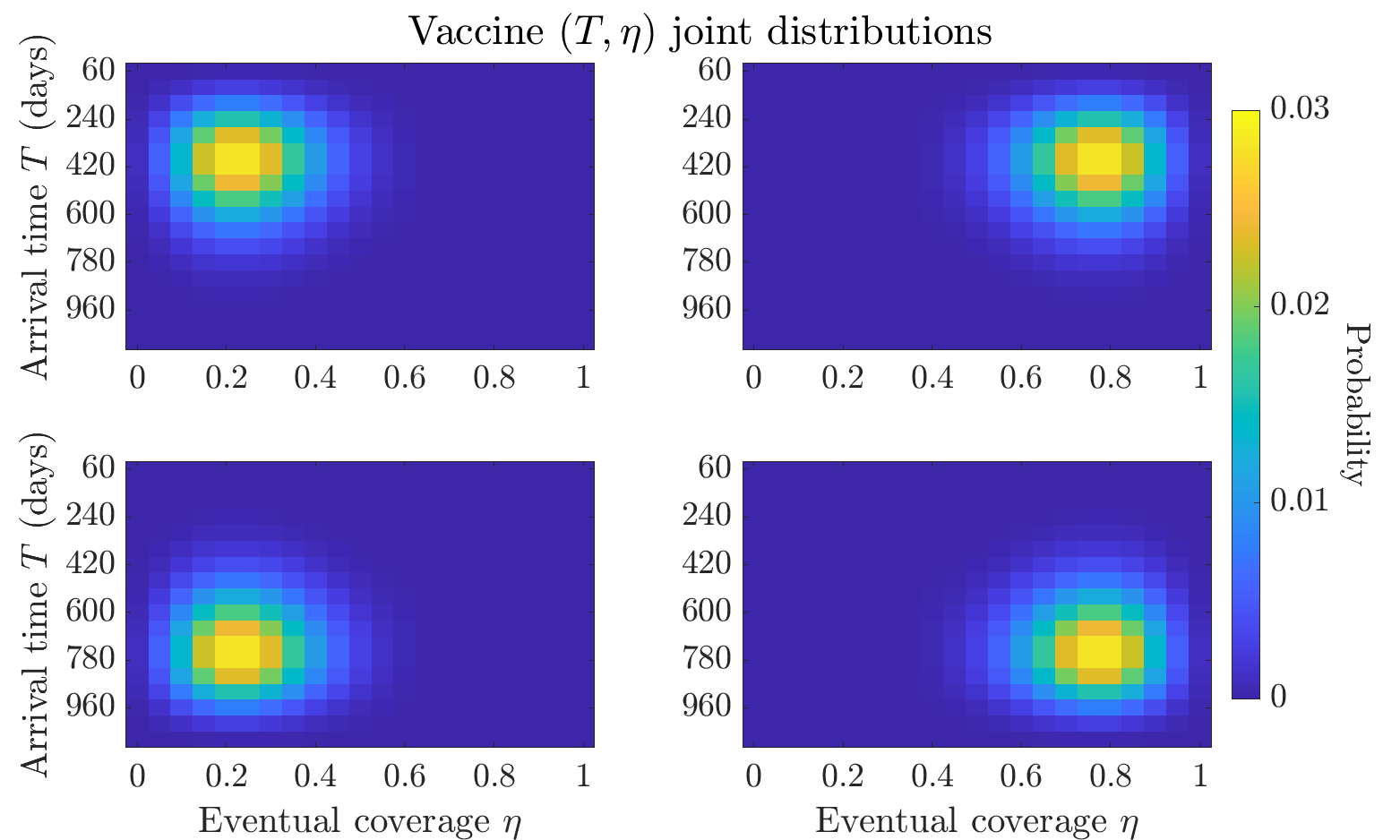

Supplement: S2 Fig — Joint probability distributions for vaccination uncertainty characterised by deployment date T and eventual coverage η. The joint distributions cover a range of optimistic (top right: low T and high η) and pessimistic (bottom left: high T and low η) scenarios regarding the effectiveness of the vaccination campaign. (TIF) [file pcbi.1012010.s004.tif]

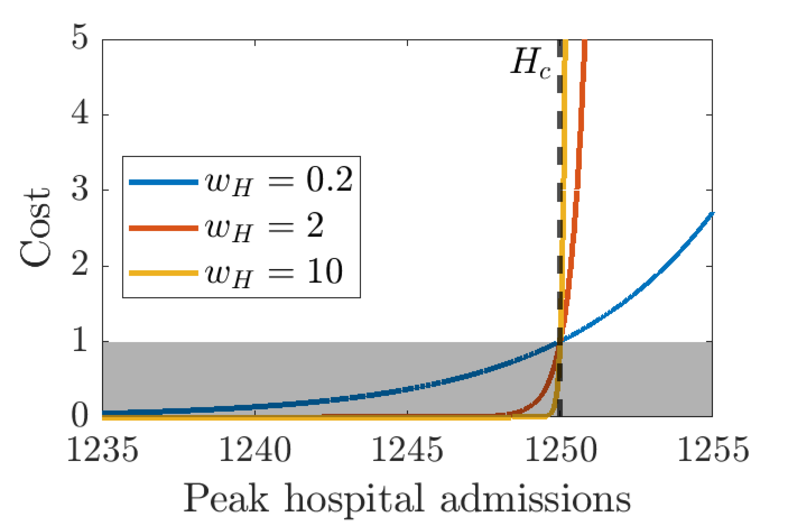

Supplement: S3 Fig — Qualitative behaviour of the exponential term in the objective function (1). Since the public health and control contributions are bounded in [0, 1] (shaded region), any excess on peak hospital capacity Hc renders the strategy infeasible with a cost exceeding at least one, regardless of weight wH. The weight wH can be scaled to deal with different perspectives regarding risk to overwhelm, but will be fixed at wH = 2 (red) for this study. (TIF) [file pcbi.1012010.s005.tif]

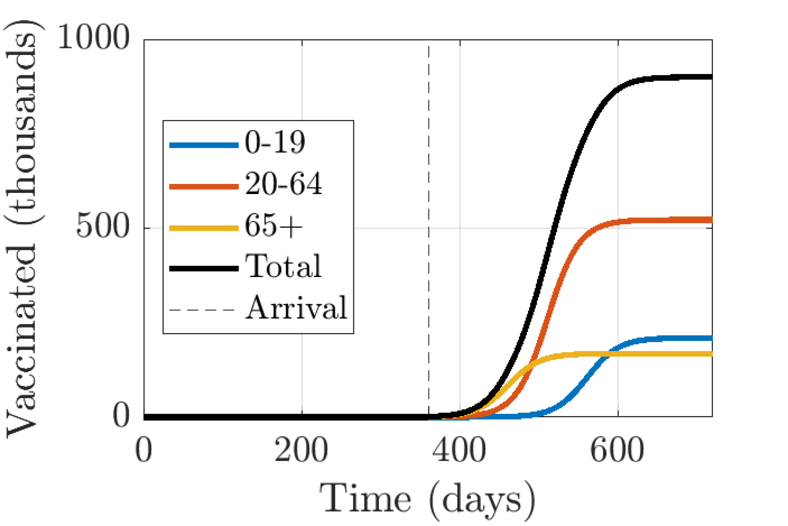

Supplement: S4 Fig — Cumulative vaccinations by age cohort Va(t) in a chosen simulation with parameter choices η = 0.9, κ = 0.05, tc = 100 days and deployment date T = 360 days. (TIF) [file pcbi.1012010.s006.tif]

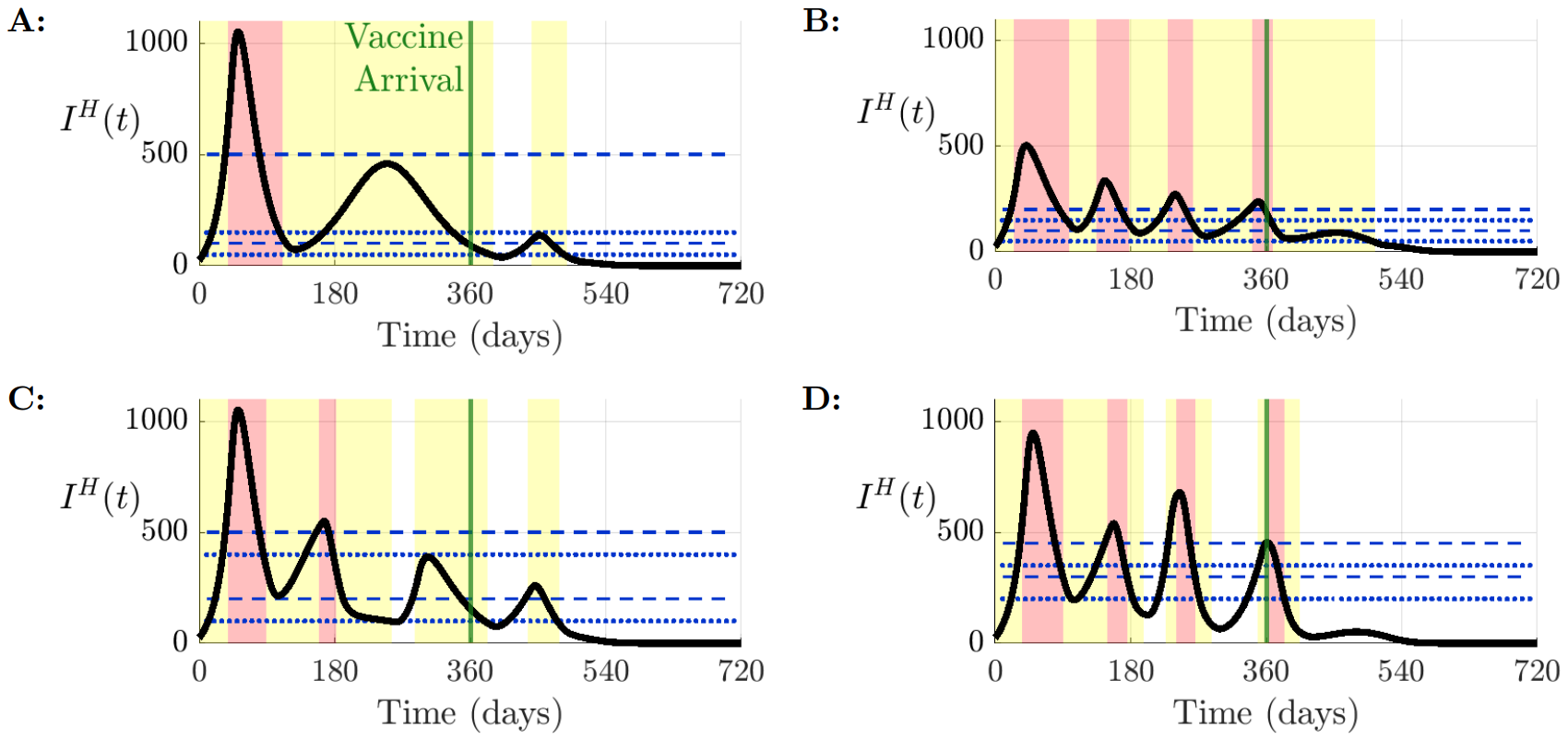

Supplement: S5 Fig — Number of active hospitalised individuals across all age cohorts IH(t) for each strategy, with a vaccine introduced on day T = 360 with η = 0.9. The periods spent in the Lockdown state and the Partial Lockdown state are shaded in red and yellow respectively. Blue dashed lines represent the reintroduction thresholds (where T12 > T01), and blue dotted lines represent the relaxation thresholds (where T21 > T10). Shaded are the periods spent in (red) Lockdown, (yellow) Partial Lockdown and (white) Inaction. Strategies are labelled as A: S1 (Cautious easing), B: S2 (Suppression), C: S3 (Slow control) and D: S4 (Rapid control). (TIF) [file pcbi.1012010.s007.tif]

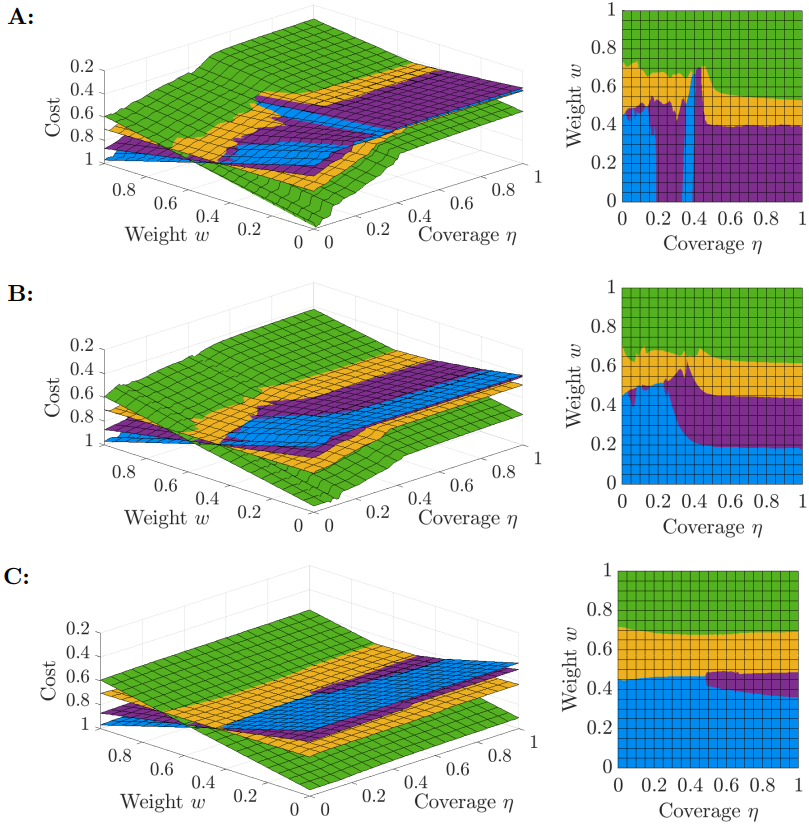

Supplement: S6 Fig — Surface plots of cost (evaluated by the objective function (1)) for different values of the weighting w and the eventual coverage of the vaccine (left). The z-axis has the lowest cost at the top, allowing the optimal strategy (corresponding to the lowest cost) to be seen more clearly. Results were generated separately for vaccine time to deployment A: T = 360, B: T = 630 and C: T = 900 days. The strategies are coloured as follows: yellow (Cautious easing), green (Suppression), purple (Slow control) and blue (Rapid control). (TIF) [file pcbi.1012010.s008.tif]

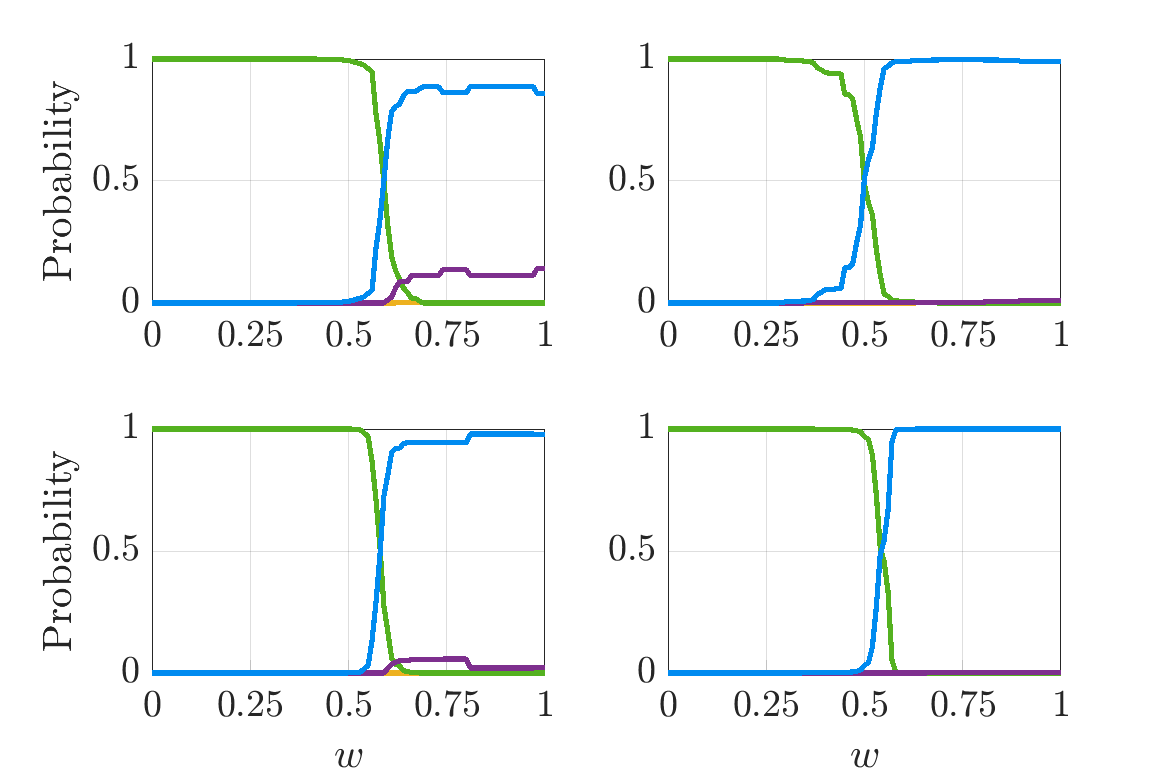

Supplement: S7 Fig — Probability that each strategy has the greatest cost according to underlying vaccine joint distribution and weight w. The joint distributions cover a range of optimistic (top right: low T and high η) and pessimistic (bottom left: high T and low η) scenarios regarding the effectiveness of the vaccination campaign. This probability is measured by ranking the strategies across all permutations of timing and coverage parameters (T, η) and summing the probabilities where each strategy is the extremum. The strategies are coloured as follows: yellow (Cautious easing), green (Suppression), purple (Slow control) and blue (Rapid control). (TIF) [file pcbi.1012010.s009.tif]

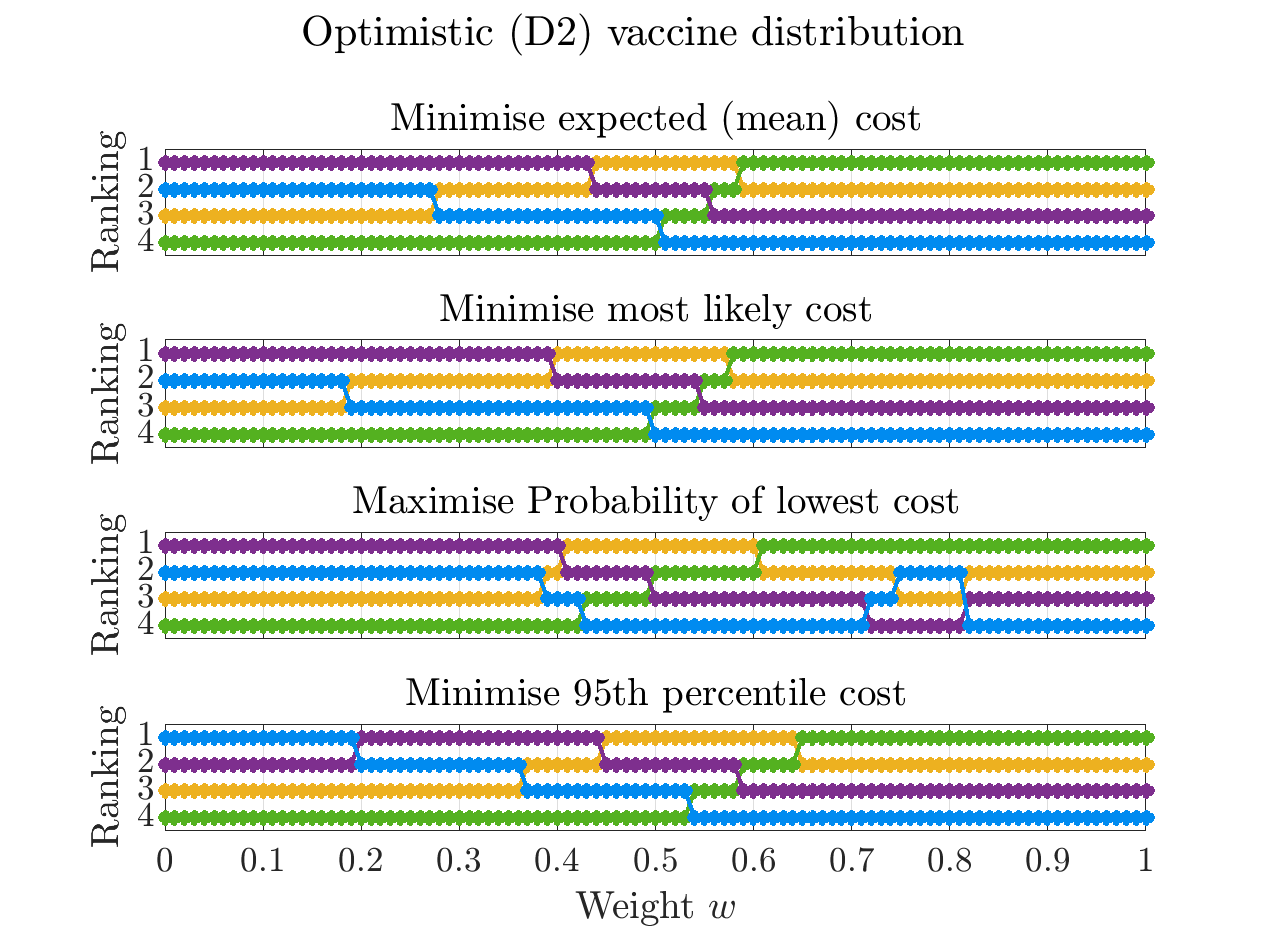

Supplement: S8 Fig — Strategy rankings according to a range of criteria described in Methods. Given a weighting w, the optimal strategy is sensitive to the summary statistic for which the policy maker seeks to minimise the objective function against. The strategies are coloured as follows: yellow (Cautious easing), green (Suppression), purple (Slow control) and blue (Rapid control). (TIF) [file pcbi.1012010.s010.tif]

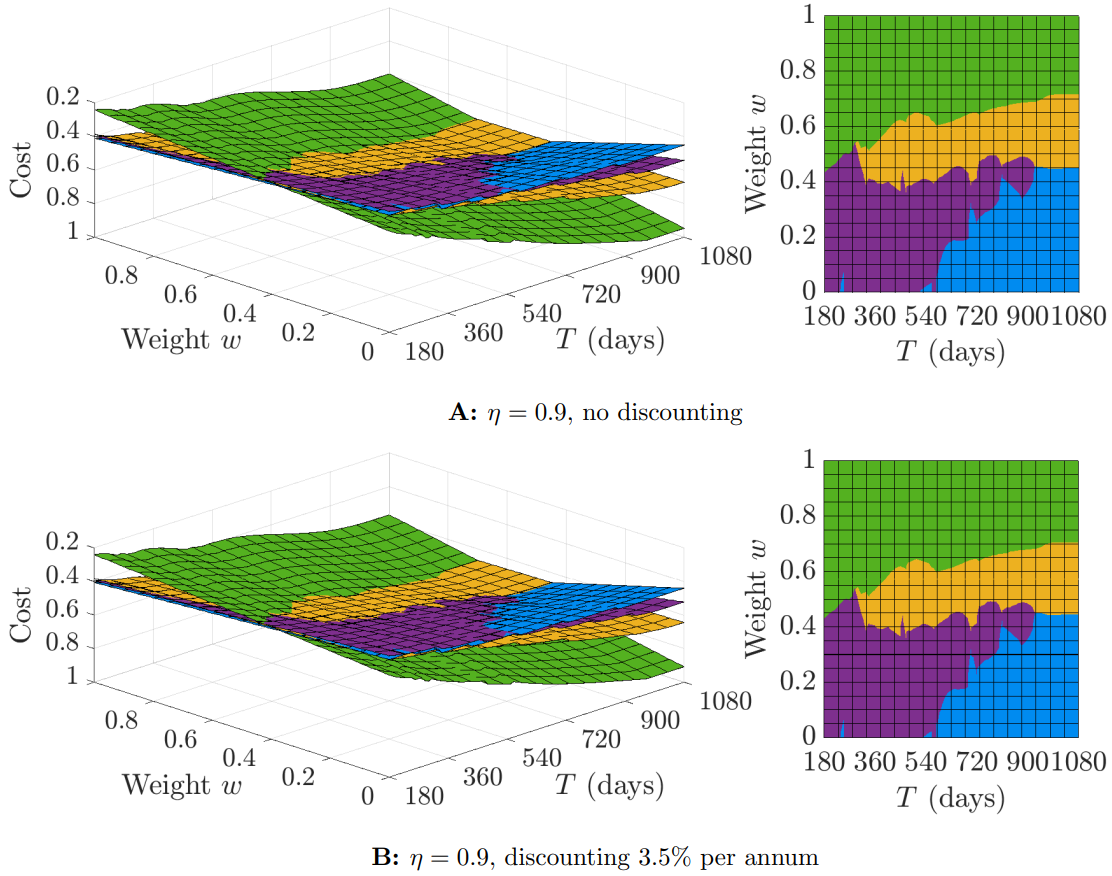

Supplement: S9 Fig — Surface plots of cost (flipped z-axis) evaluated by the objective function (1) across weighting w and different simulations which vary the arrival date of vaccination (left), and the top surface akin to viewing the optimal strategy for choice of w and vaccine arrival date (right). Simulations were separately generated for vaccine eventual coverage η = 0.9, where we apply no discounting (A, main text results) and the discounting of costs at a per-annum rate of 3.5% (B). The strategies are coloured as follows: yellow (Cautious easing), green (Suppression), purple (Slow control) and blue (Rapid control). (TIF) [file pcbi.1012010.s011.tif]
