# Supplementary material for: When should lockdown be implemented? Devising cost-effective strategies for managing epidemics amid vaccine uncertainty
Source: PLoS Comput Biol. 2024 Jul 18;20(7):e1012010. doi: 10.1371/journal.pcbi.1012010 (PMC11288439; doi:10.1371/journal.pcbi.1012010)
Supplement: Attachment — Submitted filename: PLOS-CB_response.pdf [file pcbi.1012010.s012.pdf]

May 17th, 2024

Re: PCOMPBIOL-D-24-00478. “When should lockdown be implemented? Devising cost-effective strategies for managing epidemics amid vaccine uncertainty.”

Dear Editors,

Thank you for arranging for prompt reviews of our manuscript, and for inviting us to make minor revisions. We have taken on board the reviewers' comments, which we believe we have now adequately addressed. Below are point-to-point responses to each of the reviewers’ comments.

Please note: In the material below, whenever we refer to line numbers in the revised manuscript, these refer to the version of the manuscript with “track changes”. We have uploaded two versions of the revised manuscript – one with, and one without, track changes.

Sincerely, on behalf of all authors,

Nathan Doyle

Reviewer #1

In this manuscript, Doyle and colleagues present a model-based analysis of the optimal control of an emerging epidemic from the perspective of a central policy planner. They discuss four main policies and assess how the optimal policy changes depending on the planner's beliefs about eventual vaccination coverage and timing. Rather than identifying a single optimal policy, the authors instead highlight how the planner's beliefs and priorities can impact the rankings of the available policies. Policies are assessed not just in terms of their mean expected cost, but also various other statistics including the most likely cost and the robustness (e.g., the likelihood of a worst-case scenario occurring).

The authors have chosen an appropriate model for their analysis, and they have chosen sensible simplifying assumptions (such as reducing the policy space to four main policies and considering four vaccination scenarios that span the space of plausible outcomes). Their conclusions are well supported and the paper as well written overall. My comments are minor.

Author Response: We would like to thank the reviewer for the positive appraisal of our manuscript.

1. The authors consider the optimal control strategy from the perspective of the central planner, but it is well known from the experience of COVID-19 and other epidemics that individuals also spontaneously change their behavior in response to perceived risk, sometimes reinforcing and sometimes counteracting the policies enacted by a central planner. While formally interrogating the impact of such spontaneous individual behavior is outside the scope of this manuscript, I would encourage the authors to include a section in the discussion pointing this out as a limitation of their model and highlighting some of the relevant literature on risk perception and behavior change in response to infectious disease threats.

Author Response: We agree with the reviewer that behaviour can have an impact upon the effectiveness of an intervention and therefore we have added a section to the discussion highlighting the importance of behavioural responses when considering control options. We highlight the challenges of anticipating population responses to public health messaging and incorporating formal behavioural dynamics in the infectious disease model, hence it is a limitation of our study. We had, at one point, discussed incorporating an exponential decay in intervention effectiveness in time. However, this requires us to make further assumptions (about e.g. the rate of this exponential decay), and hence we consider the question of designing optimal policy in a public health emergency with the "lockdown fatigue" phenomenon to be an area for future study. We now cite literature on the role of risk perception and spontaneous behaviour change in infectious disease modelling. The relevant section in our revised manuscript reads:

"Currently, our modelling framework does not account for spontaneous behaviour change in the population in response to the perceived risk of infection, and behavioural responses to interventions are not modelled explicitly. In practice, individuals can exhibit prevalence-elastic behaviour, i.e. the voluntary adoption of measures that limit transmission in response to an increase in the perceived infection risk as the disease becomes more prevalent [Funk et al. 2010]. On the other hand, the degree of non-

compliance with interventions in the population can change during an epidemic, particularly when measures have been introduced and relaxed several times. Such changes in population behaviour have a direct consequence on the infectious disease dynamics and thus the impact of interventions [Brooks-Pollock et al. 2023, d'Andrea et al. 2022]. Anticipating population responses to public health messaging remains a challenge, and public health interventions should be designed while considering potential resulting changes in behaviour [West et al. 2020]." (lines 284 – 296)

2. The authors discuss in the Methods that the central planner is likely to be unwilling to change policies rapidly, and therefore introduced a deterministic time horizon (two weeks) during which policies cannot be changed. It seems that it would be better to include an explicit cost of changing policies -- e.g., a cost that decays as a function of time since the most recent policy change, as an additional term in Eq (1). This way, the rate of policy change would be explicitly included in the cost model. I leave it to the editor and the authors to determine whether this is needed for the paper itself, and if not, I would just encourage the authors to more centrally discuss the fact that changing policies rapidly comes with a cost that can be quantified in a similar manner as the costs already captured by the model.

Author Response: The reviewer raises an excellent point and extending the cost function to incorporate the cost of switching policies would theoretically be possible. In this paper, however, our primary aim is to demonstrate the general principle that the optimal strategy depends on the policy maker's beliefs/priorities using a parsimonious model and cost function. Whilst a more complex cost function may more realistically capture the true costs of implementing an intervention, the disadvantage of doing this is that it would introduce at least one other variable into the model about which there is substantial uncertainty. Furthermore, for realistic incorporation of this phenomenon, such a cost could potentially be incorporated in a detailed way. For example, the cost could scale differently depending upon the control state the policy maker is switching to – it is surely less costly to switch back to the n-th No Control as opposed to switching back to the n-th Lockdown. We therefore believe that exploring the optimal strategy with a cost function which penalises frequent switching (via a structure suggested by the reviewer or as a constraint) is an area that we would be keen to prioritise for future work, but that – after substantial reflection – we prefer to maintain our simple assumption for this initial study. We now highlight this area for further consideration in the discussion, noting that modelling for policy needs to consider that policy makers must balance the need to implement interventions rapidly to curb exponential growth, whilst acknowledging the

reluctance of policy makers to change policy frequently. The relevant section in our revised manuscript reads:

"In this study, we imposed a constraint on the minimum duration spent in each control state to reflect the fact that policy makers are typically unwilling to change public health measures multiple times in quick succession. However, the act of implementing or removing an intervention may come with a quantifiable cost. This could be incorporated into our modelling framework by adapting the current objective function, to penalise policies that switch between control states frequently. Such an extension could take the form of a sunk cost for switching into a new control state, or a cost that decays as a function of time between policy changes." (lines 272 – 280)

Reviewer #2

Main comments

I appreciated that in this study, the authors used the motivation of the COVID-19 pandemic, to then present a modelling framework for informing future decisions that is more broadly applicable beyond just COVID – and I suppose there is a bit of a balancing act between specificity and generalisability here. However, I thought that one component that was potentially overlooked here was the model structure itself. To what extent do you think the form of the compartmental age-structured SEIRS-type model selected for this analysis (where the model really applied to COVID) might have changed the results? I'm not suggesting you need to explore other model structures as that would be beyond the scope of the current study but I think worth commenting on how this study is restricted somewhat by the model structure chosen, and that other model structures are possible (e.g. for a vector-born disease where the compartmental structure may look different?).

Author Response: Thank you for noting this. It is an important point; we agree that the model structure is inherent from our COVID-19 motivation. We have added some discussion, highlighting that the precise model to use to guide decision making is context dependent. In principle, our methodology can be applied using a range of different model structures. Nonetheless, we contend that the takeaway message of the manuscript - that the optimal strategy depends on a policy maker's beliefs/priorities - would not be affected by the structure of the model used. We agree that applying our approach to different models is out of scope for this manuscript, but it is an important area for future work. The relevant section in the discussion in our revised manuscript reads:

“We also acknowledge that the predicted effectiveness of different interventions will depend on the underlying model used to simulate their impacts. The model structure used in this study was motivated by compartmental models used during the COVID-19 pandemic [Keeling et al, 2022], but other model architectures and settings are possible. Before mathematical models can be used to inform policy, one should carefully consider (i) the appropriate model structure to match the epidemiology of the pathogen driving the outbreak under consideration and (ii) the precise control strategies to consider (limiting this choice to those that can be applied in practice). Extending our approach to consider a range of different compartmental models suitable for modelling a range of pathogens is an area for future research.” (lines 307 – 316)

In the methods, I found the section on “Control” hard to follow – and I think it is to do with the notation and terminology. You explain the three states of “No Control”, “Intermediate Control” and “Lockdown”, and the levels of transmission reduction associated with these (fine). I think these are subsequently referred to as “control states”. But then you talk about “different control policies”, and then “four control strategies” (line 350). As far as I could understand, the control strategies place different thresholds on switching between control states. And then on line 380 you mention “control measures”. I think it is just all the different version of control being used that is a little hard to follow (I had to read it a couple of times to make sure I understood). You may want to consider the terminology more carefully. In Table 1, I also think a bit more detail could be provided in the column headings and the caption.

Author Response: We thank the reviewer for this valid comment, and after revising we understand how the current wording might not be as clear as it can be. We have modified the manuscript to make our terminology of “control” as clear as possible. We maintain the use of the term “control states” to refer to the three levels of interventions a policy maker can move between to mitigate the outbreak. We have renamed the “No Control” and “Intermediate Control” states to “Inaction” and “Partial Lockdown” respectively. The four “control strategies”, which are defined by the precise hospital occupancy thresholds for switching between the three control states, are now solely referred to as “strategies”. We have elaborated upon the caption and column headings in Table 1.

Minor comments

How were the 40% and 70% levels of reduction in transmission for the control scenarios selected?

Author Response: The 70% reduction in transmission for the Lockdown state is informed by the basic reproduction number of $R_0 = 3$, which means that at most two-thirds of transmission events need to be prevented to bring the real-time reproduction number under 1. In practice, it should be an ensemble of social distancing measures which are sufficient to bring the outbreak under control. In contrast, the Partial Lockdown state (previously named "Intermediate Control", see response directly above) is designed to slow the outbreak's spread but is ultimately insufficient to reverse it. This does not account for the depletion of susceptibles, which is why Partial Lockdown can bring the outbreak under control in later stages (Figure 1C,1D).

I found it really hard to interpret Figure 1 – the switching thresholds indicated by the dashed lines didn't seem to line up with T_{01}, T_{12}, and so on, on the right-hand side axis?

Author Response: Figure 1 has been re-formatted to try to make this clearer. The caption has been altered to aid explanation: "Blue dashed lines represent the reintroduction thresholds (where $T_{12} > T_{01}$), and blue dotted lines represent the relaxation thresholds (where $T_{21} > T_{10}$)." Similar changes have been applied to Figure S5 (hospitalisation dynamics in a vaccination scenario) and its corresponding caption. (lines 574 – 580)

For Figure 2 – what is the difference between the panels in the left column versus the right column?

Author Response: The initial caption explains the difference between the left and right subpanels: "The figure shows the scenario of no vaccination (left) contrasted with a vaccination scenario for $\eta = 0.9$ and $T = 360$ days (right)". I have now added titles to the subpanels in Figure 2A to make this clearer.

Line 145-146, where you state that in the model, the value of the weight w relates to the policymakers choice about costs of disease versus costs of public health measures – would be helpful to explain here what a low versus high value of w means to aid intuition for the reader.

Author Response: Thank you for pointing this out, the following clarification has been added to the manuscript:

"For high values of w, the policy maker favours the reduction of cumulative hospitalisations in the objective function, whereas for low values of w their priority

switches to reducing the cost of maintaining interventions (see objective function (1)).” (lines 152 – 155)

Objective function. Line 401 onwards – did you explain here what w_H is?

Author Response: We explain by adding the following:

“The rate at which strategies with epidemic peaks beyond or near capacity are penalised is controlled by the parameter w_H. This component gives a negligible contribution to strategies which maintain a below-capacity level of hospitalised individuals, but a rapidly increasing contribution once hospital capacity is exceeded” (lines 460 – 464)

Figures S10 to S15 – it’s not clear what values of the parameters being varied are applied in each of the panels within these figures.

Author Response: Additional detail has now been added describing how the sensitivity analysis was conducted, and which parameter values are being varied (see S1 Text, Sensitivity Analysis and Figures S10 – S19).

Line 60. “when designing models for use to guide interventions”

Author Response: This sentence has been revised to read the following:

“A key challenge for epidemiological modellers when designing models to guide interventions is to account for uncertainties about outbreak dynamics and the effects of those interventions, particularly in the early stages of the outbreak.” (lines 62 – 64)
